# Artemisinin conferred cytoprotection to human retinal pigment epithelial cells exposed to amiodarone-induced oxidative insult by activating the CaMKK2/AMPK/Nrf2 pathway

**DOI:** 10.1186/s12967-024-05593-x

**Published:** 2024-09-16

**Authors:** Chao Yang, Xia Zhao, Wenshu Zhou, Qin Li, Philip Lazarovici, Wenhua Zheng

**Affiliations:** 1 Pharmaceutical Science, Faculty of Health Sciences, University of Macau, Taipa, Macau SAR 999078 China; 2 Ministry of Education Frontiers Science Center for Precision Oncology, University of Macau, Taipa, Macau SAR 999078 China; 3 Pharmacology, School of Pharmacy Institute for Drug Research, Faculty of Medicine, The Hebrew University of Jerusalem, Jerusalem, Israel

**Keywords:** Artemisinin, Amiodarone, Human retinal pigment epithelial cells, Oxidative damage, AMPK, Ocular toxicity

## Abstract

**Background:**

Ocular toxicity is a severe adverse effect that limits the chronic clinical use of the antiarrhythmic drug amiodarone. Here, we aimed to evaluate the cytoprotective effect of artemisinin and explore the potential signalling pathways in human retinal pigment epithelial (RPE) cell cultures.

**Methods:**

D407 cell cultures were exposed to amiodarone and the impact of artemisinin was evaluated. The key parameters included lactate dehydrogenase (LDH) release, intracellular reactive oxygen species (ROS) generation, and the mitochondrial membrane potential (MMP). We also assessed the protein levels of cleaved caspase-3, cleaved poly (ADP-ribose) polymerase (PARP), phosphorylated adenosine monophosphate-activated protein kinase (AMPK)ɑ (p-AMPK), calcium/calmodulin-dependent protein kinase kinase 2 (CaMKK2), and nuclear factor erythroid 2-related factor 2 (Nrf2).

**Results:**

Artemisinin reduced the cytotoxicity induced by amiodarone, as reflected by decreased LDH release, ROS generation, and MMP disruption. Additionally, artemisinin increased p-AMPK, CaMKK2, and Nrf2 protein levels. Inhibition of AMPK, CaMKK2, or Nrf2 abolished the cytoprotective effect of artemisinin. AMPK activation and Nrf2 knockdown further supported its protective role.

**Conclusions:**

Artemisinin protected RPE cells from amiodarone-induced damage via the CaMKK2/AMPK/Nrf2 pathway. The in vivo experiments in mice confirmed its efficacy in preventing retinal injury caused by amiodarone. These results suggest that an artemisinin-based eye formulation could be repurposed for treating amiodarone-induced ocular toxicity.

**Supplementary Information:**

The online version contains supplementary material available at 10.1186/s12967-024-05593-x.

## Background

Amiodarone is a class III antiarrhythmic drug indicated for the treatment of recurrent haemodynamically unstable ventricular tachycardia and ventricular fibrillation. However, upon chronic treatment, amiodarone has serious adverse effects on many organs such as the eyes, lungs, thyroid, liver, skin, and nerves, so it is generally considered a secondary therapeutic option [1]. Amiodarone-induced ocular toxicity is the most common adverse event and includes corneal micro-deposits [2], anterior sub-capsular lens deposits, and optic neuropathy [3, 4]. The characteristics of amiodarone-associated optic neuropathy include an insidious onset, slow progression, bilateral visual loss, and optic disc edema [5]. The exact cause of amiodarone-related optic neuropathy is still unknown [4]. Although optic disc edema resolves and visual acuity improves after the use of AM is stopped, some patients may still be permanently blind, and there is currently no cure for blindness [3, 5–8].

The retina is a thin layer of tissue that lines the inner side of the back of the eye. The main function of the retina is to receive the light focused by the lens and convert it into neural signals, and then the optic nerve sends these signals to the brain for visual recognition [9]. The retinal pigment epithelial (RPE) layer is a single layer composed of cubic epithelial cells that regulate the transport of nutrients, metabolic products, and ions to the neural retina [10]. The melanin in RPE cells prevents light from being reflected to the entire eyeball, which is important for clear vision [11]. Previous research has shown that in the mouse retinal neuronal cell line RGC-5 and the human RPE cell line D407, the cytotoxicity caused by amiodarone involves several intracellular events, including oxidative stress, mitochondrial dysfunction, caspase activation, and apoptosis [12, 13].

Artemisinin is a well-known antimalarial drug extracted from the plant *Artemisia annua* L and has been used in the clinic for decades [14]. It is safe and economical and can easily cross the blood-brain barrier. ART has been shown to protect against H_2_O_2_-induced oxidative injury and apoptosis in human RPE cells, bone marrow-derived mesenchymal stem cells, human neuroblastoma cells, and hippocampal neurons [15–18]. However, whether artemisinin can inhibit amiodarone-induced oxidative stress and apoptosis in human RPE cells is unknown.

Adenosine monophosphate-activated protein kinase (AMPK) is an evolutionarily conserved cellular energy sensor that exists in mammals, yeast, and plants and is composed of three subunits, ɑ, β, and γ. AMPK activation is reflected by increased phosphorylation level of the AMPK ɑ-subunit (AMPKɑ) at Thr172. The activation of AMPK plays an essential role in preventing the degeneration of photoreceptors and RPE cells [19–21]. Compared with the AMPKɑ1(PRKAA1) protein, the AMPKɑ2 (PRKAA2) protein has been reported to have an important cytoprotective effect on RPE cells and retina [21, 22]. Therefore, we focused on AMPKɑ2 in this study. Calcium/calmodulin-dependent protein kinase kinase 2 (CaMKK2) and liver kinase B1 (LKB1) are two essential upstream kinases of AMPK, and are involved in AMPK activation [23–26]. It has been reported that the artemisinin-mediated protection of RPE cells against H_2_O_2_-induced oxidative injury is dependent on AMPK activation [16]. Therefore, the present study further explores this hypothesis by focusing on drug-induced oxidative stress and AMPK.

The nuclear factor erythroid 2-related factor 2 (Nrf2)/heme oxygenase-1 (HO-1) pathway is a crucial mechanism that defends cells against oxidative stress, which is caused by the accumulation of reactive oxygen species (ROS) [27]. In the retina, this pathway is essential for maintaining the health and function of retinal cells. By detoxifying ROS, the Nrf2/HO-1 pathway helps reduce inflammation and prevent cell death in the retina [27, 28]. This phenomenon is particularly important in retinal diseases characterized by oxidative stress, such as age-related macular degeneration (AMD) and uveitis [27, 28].

We found that artemisinin conferred cytoprotection against amiodarone-induced oxidative stress and apoptosis in human RPE cells in correlation with CaMKK2/AMPK/Nrf2 activation. This finding may be exploited for the formulation of artemisinin, and for repurposing its use in the clinic for the treatment of amiodarone-induced ocular toxicity.

## Materials and methods

### Chemicals

MTT was acquired from Molecular Probes (Eugene, OR, USA). Amiodarone was purchased from Sanofi Winthrop Industrie (Ambarès-et Lagrave, France). Artemisinin was obtained from Meilun (Dalian, China). The pancaspase inhibitor Z-VAD-FMK was purchased from Calbiochem (San Diego, CA, USA). The AMPK inhibitor Compound C and the AMPK activator 5-aminoimidazole-4-carboxamide ribonucleotide (AICAR) were purchased from Selleckchem (Houston, Texas, USA). The CaMKK2 inhibitor STO-609 was purchased from Selleckchem (Houston, Texas, USA). The Nrf2 inhibitor ML385 was acquired from Signalway Antibody (SAB, College Park, Maryland, USA).

### Cell culture

D407 (human RPE cell line) cells obtained from the cell bank, at Sun Yat-sen University (Guangzhou, China) were cultured in DMEM (GIBCO, Grand Island, NY, USA). ARPE19 (human RPE cell line) cells purchased from Shanghai Kanglang Biotechnology Co., Ltd. (Shanghai, China), and primary human RPE cells provided by the State Key Laboratory of Ophthalmology, Zhongshan Ophthalmic Center, Sun Yat-sen University, were cultured in DMEM-F12 (GIBCO). Both DMEM and DMEM-F12 contained 100 units/mL penicillin, 100 µg/mL streptomycin (GIBCO), and 10% FBS (GIBCO). Cell cultures were grown at 37℃ in the incubator with 5% CO_2_ humidified atmosphere.

### MTT assay

D407 cells, ARPE19 cells, or primary human RPE cells were seeded in 96-well plates (4 ∼ 6 × 10^3^ cells/well) and grown for at least 16 h. The cells were treated with drugs diluted in the FBS-free medium and then incubated with 0.5 mg/mL MTT for another 3 h. Thereafter, the medium was aspirated, and DMSO (100 µl/well) was added to the wells. The optical density (OD) value was detected at 570 nm wavelength using a microplate spectrophotometer (Thermo Fisher Scientific, Waltham, MA, USA) [16].

### Lactate dehydrogenase (LDH) cytotoxicity assay

LDH can leak into the culture medium from cells with ruptured plasma membranes caused by apoptosis or necrosis, and LDH activity can be detected to measure the cytotoxicity. D407 cells and primary human RPE cells were seeded in 96-well plates (5 × 10^3^ cells/well) and grown for at least 16 h. The cells were treated with drugs diluted in the FBS-free medium, after which the LDH activity in the cell culture supernatant was detected using a LDH cytotoxicity assay kit (Beyotime, Shanghai, China). The OD value was detected at a wavelength of 490 nm using a Victor X3 microplate reader (PerkinElmer, Waltham, MA, USA).

### ROS assay

D407 cells attached to 96-well plates (5 × 10^3^ cells/well) were treated with or without amiodarone in the absence or presence of artemisinin for 24 h and then stained for 40 min with DCFH-DA (Beyotime, Shanghai, China). ROS level was measured by detecting the green fluorescence intensity using a Victor X5 microplate reader (PerkinElmer, Waltham, MA, USA). Green fluorescence was observed with an EVOS™ M7000 imaging system (Thermo Fisher Scientific, Waltham, MA, USA) (20× magnification).

### Mitochondrial membrane potential (MMP) assay

D407 cells and primary human RPE cells attached to 96-well plates (5 × 10^3^ cells/well) were treated with or without amiodarone in the absence or presence of artemisinin for 24 h and then stained for 40 min with JC-1 dye (Beyotime, Shanghai, China). Green and red fluorescence was observed by an EVOS™ M7000 imaging system (Thermo Fisher Scientific, Waltham, MA, USA) (20× magnification). The green fluorescence and red fluorescence intensities were measured with a Victor X5 microplate reader. The MMP was measured by determining the red/green fluorescence ratio [16].

### Western blotting

D407 cells attached to 12-well plates (1.5 ∼ 2.5 × 10^5^ cells/well) and ARPE19 cells attached to 24-well plates (2.5 × 10^5^ cells/well) were treated with drugs and lysed with lysis buffer on ice. The lysates were boiled for 15 min and cooled, after which the protein concentration was determined using the bicinchoninic acid (BCA) method. Protein samples were separated by 4 ∼ 20% SurePAGE™ precast gels (GenScript, New Jersey, USA) (80 V, 20 min; 120 V, 70 min) and then transferred onto PVDF membranes (90 V, 120 min). After transferring, the membranes were blocked with 5% (w/v) nonfat milk in TBST buffer for 1 h. After washing, the membranes were incubated with the respective primary antibody solution at 4℃ overnight, and then incubated with the secondary antibody solution at room temperature for 1 ∼ 2 h. After washing, the Clarity Western ECL Substrate (Bio-Rad, Hercules, CA, USA) was used to visualize the protein bands. Image J software was used for the quantification analysis of protein bands [29].

The antibody name, type, source, dilution, and company are shown in Table 1.

**Table 1:** List of antibodies used for Western blotting in this study

Antibody name	Clonality	Source	Dilution	Company (Catalog Number)
GAPDH	Monoclonal	Rabbit	1:1000	CST (3683)
Phospho-AMPKɑ (Thr172)	Monoclonal	Rabbit	1:1000	CST (2535)
Phospho-AKT (Ser473)	Polyclonal	Rabbit	1:1000	CST (9271)
Nrf2	Monoclonal	Rabbit	1:1000	CST (12721)
HO-1	Monoclonal	Rabbit	1:1000	CST (5853)
PARP/cleaved PARP	Polyclonal	Rabbit	1:1000	CST (9542)
Cleaved caspase-3	Monoclonal	Rabbit	1:1000	CST (9664)
AMPKɑ2	Polyclonal	Rabbit	1:1000	CST (2757)
CaMKK2	Polyclonal	Rabbit	1:1000	SAB (43469)
LKB1	Polyclonal	Rabbit	1:1000	SAB (24473)
Anti-rabbit IgG HRP-linked secondary antibody	Polyclonal	Goat	1:2000	CST (7074)

CST: Cell Signaling Technology (Woburn, MA, USA); SAB: Signalway Antibody (College Park, Maryland, USA)

### Plasmid transfection

The human Nrf2 short hairpin RNA (shRNA) plasmids (target sequence: GGTTGAGACTACCATGGTTCC) and control shRNA plasmids (CON313, target sequence: TTCTCCGAACGTGTCACGT) were obtained from GenPharma Co., Ltd. (Shanghai, China). The human AMPKɑ2 shRNA plasmids (target sequence: TGTGAAAGAAGTGTGTGAA), control shRNA plasmids (CON207, target sequence: TTCTCCGAACGTGTCACGT), the AMPKɑ2-overexpressing plasmids, and control overexpressing plasmids (CON238) were purchased from GenePharma Co., Ltd. (Shanghai, China). D407 cells were seeded in 12-well plates (1 ∼ 2 × 10^5^ cells/well) or 24-well plates (5 × 10^4^ cells/well). On the second day, the medium was changed without FBS, and the cells were transfected with control plasmids, human AMPKɑ2/Nrf2 knockdown plasmids, or human AMPKɑ2 overexpression plasmids using Lipofectamine 3000 transfection reagent (Thermo Fisher Scientific, Waltham, MA, USA) following the manufacturer’s instructions. Cellular proteins harvested from 12- or 24-well plates were used to confirm the knockdown/overexpression efficiency via western blotting [30].

### Animals and treatment

Male BALB/c mice (6 weeks) were reared and maintained according to institutional guidelines and randomly divided into four groups (eight mice per group), and the weight of each group of mice was between 19 ∼ 21 g. After the mice were anesthetized, 0.5% tetracaine eye drops were used for corneal anesthesia, and tropicamide was used to dilate the pupils. A 5 µl microsyringe was inserted at the posterior limbus of the superior temporal quadrant, and the drug solution or PBS was injected into the vitreous body. In Group 1 (the sham control group), the mice were given an intraperitoneal injection of 200 µl of PBS and a vitreous injection of 2 µl of PBS in each eye. In Group 2 (the amiodarone-treated model group), the mice were given an intraperitoneal injection of 200 µl of PBS and a vitreous injection of 3 µM amiodarone (2 µl per eye). In Group 3 (the artemisinin-treated amiodarone model group), the mice were given an intraperitoneal injection of artemisinin (10 mg/kg) 1 h before vitreous injection of amiodarone. In Group 4 (the Compound C and artemisinin combination-treated amiodarone model group), the mice were given an intraperitoneal injection of Compound C (5 mg/kg) 1 h before the injection of artemisinin compared to those in Group 3 [31].

### Electroretinography (ERG) assay

One week after the injection of amiodarone, a focal ERG assay was performed to assess the function of the retinal tissue. Photopic 3.0 ERG of the mice was recorded by using an animal electrophysiology vision system (Roland, Brandenburg, Germany). The ERG waveform represents the electrical response of the entire retina when it is subjected to a brief light stimulus [32]. The first large negative component is the a-wave, which is produced by cone photoreceptors in the outer nuclear layer (ONL) of the retina. The lowest point on the curve is manually defined as the trough of the a-wave. The large positive component is the b-wave, which is produced by bipolar cells in the inner nuclear layer (INL) of the retina. The highest point on the curve is manually defined as the peak of the b-wave [12].

### Hematoxylin-eosin (HE) histological staining

Following the focal ERG assay, the eye tissues were removed. After being fixed and dehydrated, the tissues were embedded in paraffin. The samples were sectioned (5 μm), and HE staining was performed via a ST5020 Multistainer (Leica, Buffalo Grove, USA). The sections were scanned with a NanoZoomer S60 digital slide scanner (Hamamatsu, Japan), and histopathological changes in mouse retinas were analysed via NDP.view2 viewing software (Hamamatsu, Japan) (20× magnification).

### TdT-mediated dUTP nick-end labelling (TUNEL) assay

After the deparaffinization of eye tissue Sect. (5 μm), the apoptotic cells in the retina of the eye sections were detected via a one step TUNEL apoptosis assay kit (Beyotime, Shanghai, China) according to the manufacturer’s instructions. The fluorescence was observed, and images were obtained using an EVOS™ M7000 imaging system.

### Data analysis

Data analysis was performed using GraphPad Prism 5.0 statistical software (San Diego, CA, USA), and the results were expressed as the mean ± SEM (*n* ≥ 3). The unpaired two-tailed t-test was utilized when two conditions were compared. Statistical significance was determined at * *p* < 0.05, ** *p* < 0.01, and *** *p* < 0.001. “ns” means not significant (*p* > 0.05).

## Results

### Artemisinin inhibited the amiodarone-induced decrease in cell viability, release of LDH, generation of intracellular ROS, and decrease in the MMP in D407 cell cultures

**Fig. 1:**
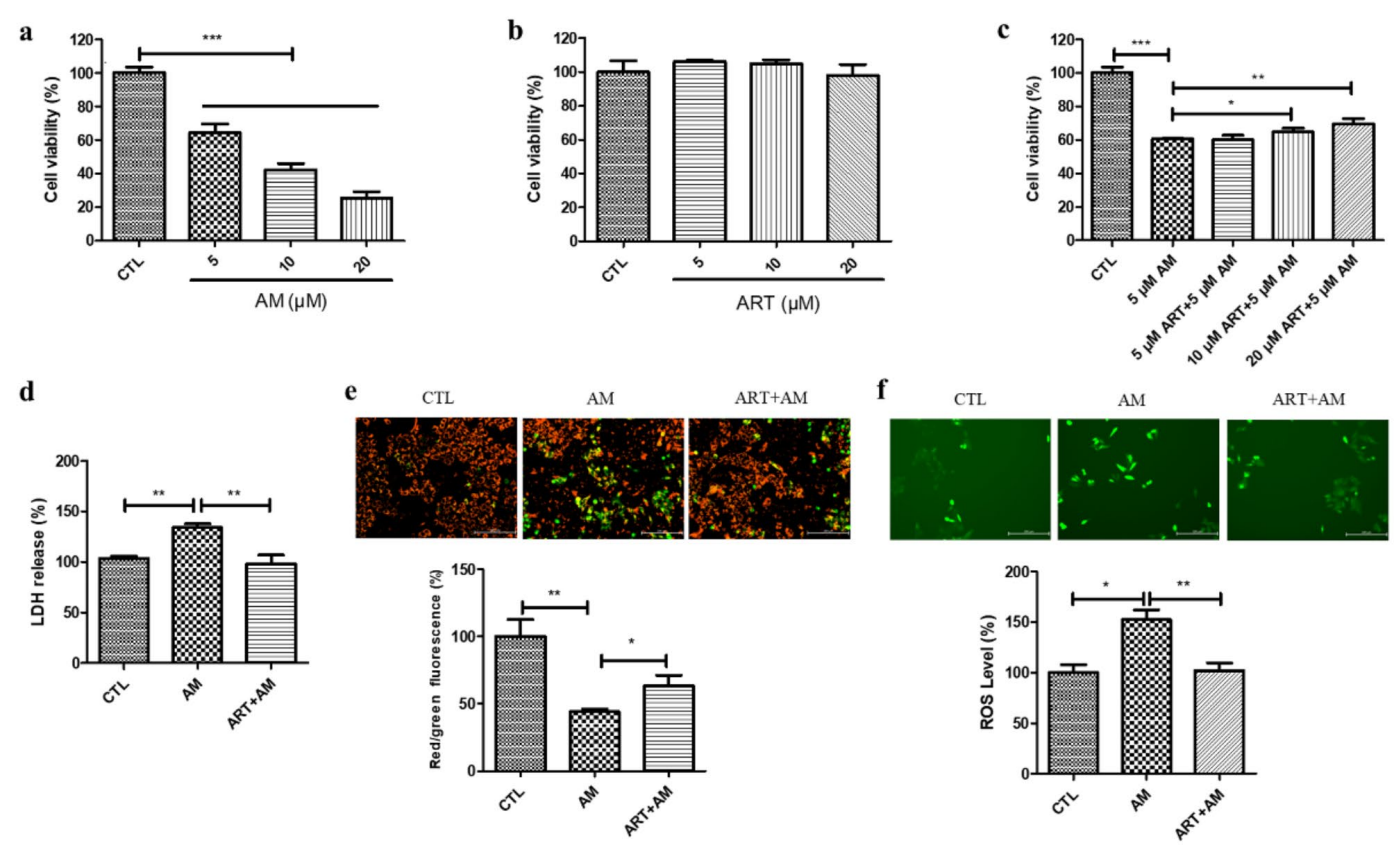
Artemisinin protected D407 cells from the decrease in viability, increase in LDH release, intracellular ROS generation, and decrease in the MMP induced by amiodarone. (**a-b**) Cells attached to 96-well plates were incubated with different concentrations of amiodarone (AM) or artemisinin (ART), and cells incubated with 0.1% DMSO were used as the control (CTL) group. Twenty-four hours later, cell viability was determined through the MTT assay (*n* = 4). (**c**) The cells were pretreated with or without artemisinin (5, 10, or 20 µM) for 1 h and then incubated with 5 µM amiodarone for another 24 h. Cell viability was determined through the MTT assay (*n* = 3). (**d-f**) The cells were pretreated with or without 20 µM artemisinin for 1 h and then incubated with 5 µM amiodarone for another 24 h. Cell death was measured by LDH release (*n* = 3). The MMP was measured using JC-1 dye (*n* = 3). The ROS level was measured using the probe DCFH-DA (*n* = 4). Red and green fluorescence was observed under a fluorescence microscope, and the fluorescence intensity was quantified using a fluorescence microplate reader. The scale bar is 200 μm

The MTT assay results using D407 cell cultures showed that treatment with 5, 10, or 20 µM amiodarone caused a decrease in cell viability compared with that of the control group (Fig. 1a). Treatment of D407 cell cultures with 5, 10, or 20 µM artemisinin did not influence cell viability (Fig. 1b). To determine the effect of artemisinin on the amiodarone-induced decrease in cell viability, D407 cells were pretreated with artemisinin for 1 h before exposure to amiodarone. Figure 1c showed that, in a concentration-dependent manner, artemisinin pretreatment gradually attenuated the amiodarone-induced decrease in cell viability by 4% or 9%.

The results of the cytotoxicity assessment further support the results of the MTT assay. Compared with the control treatment, incubation of D407 cells with 5 µM amiodarone for 24 h induced the release of LDH in the medium, indicating increased cell death. Pretreatment with 20 µM artemisinin for 1 h significantly inhibited the amiodarone-induced release of LDH by 37% (Fig. 1d). The results of the MMP assay revealed that, compared with that in the control group, the green fluorescence in the amiodarone-treated group increased, whereas the red fluorescence in the amiodarone-treated group decreased, implying a decrease in the MMP and early apoptotic events. Pretreatment with 20 µM artemisinin for 1 h significantly attenuated the decrease in the MMP by 19% (Fig. 1e). The results of the ROS assay revealed that the green fluorescence in the AM-treated group was greater than that in the control group, indicating that oxidative stress was caused by an increase in the intracellular ROS level. Pretreatment with 20 µM artemisinin for 1 h reduced the amiodarone-induced increase in the ROS level by 50% (Fig. 1f). Cumulatively, these results indicate that artemisinin conferred cytoprotection as revealed by improvement in cell viability, decrease in LDH release, attenuation of intracellular ROS, and correction of the decrease in the MMP in D407 cell cultures.

### Artemisinin inhibited the activation of proapoptotic proteins caused by amiodarone in D407 cell cultures

**Fig. 2:**
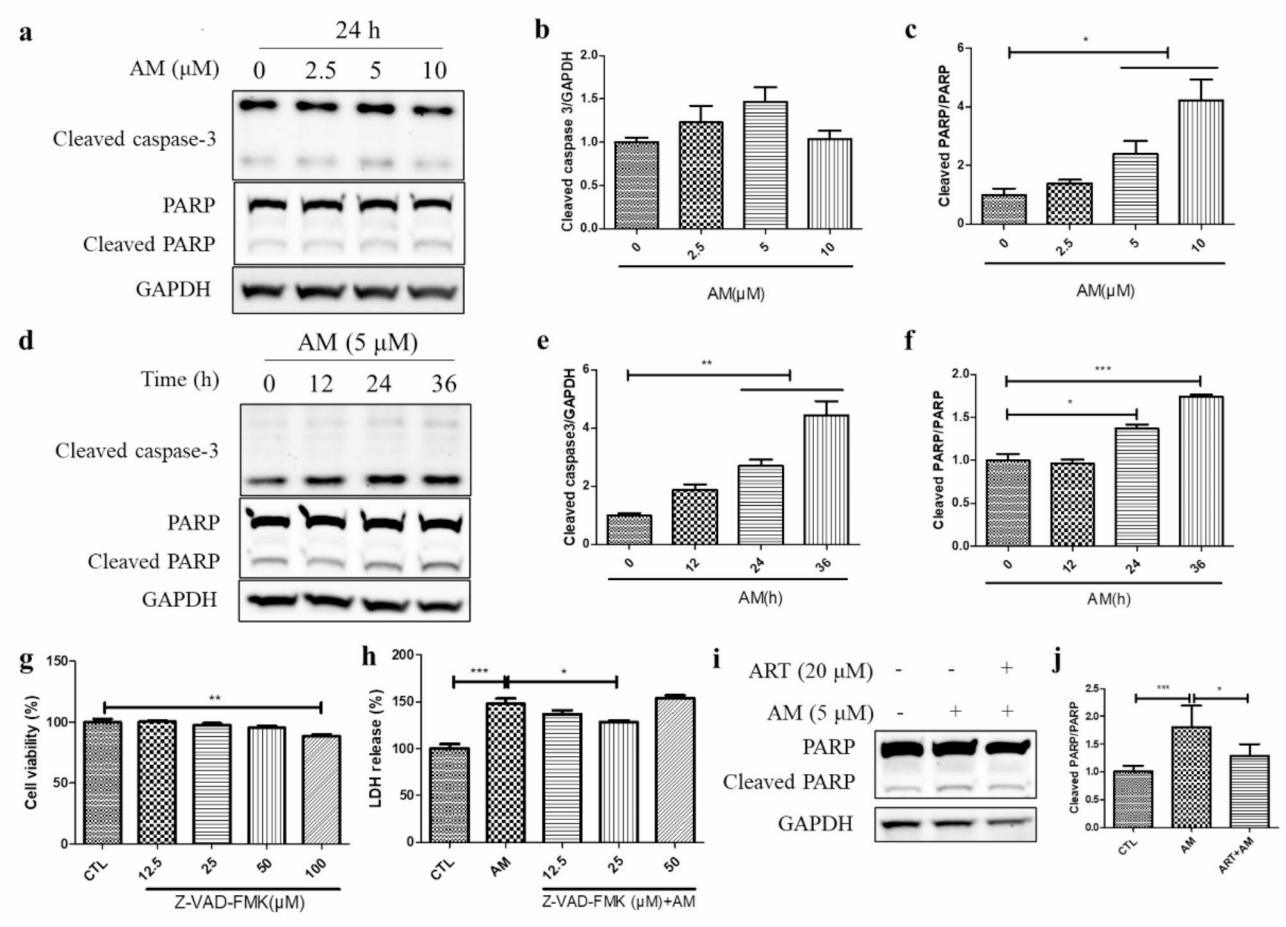
Artemisinin protected D407 cell cultures from the amiodarone-induced activation of proapoptotic proteins. (**a-c**) Cells attached to 12-well plates were incubated with different concentrations of amiodarone (2.5, 5, or 10 µM) for 24 h. The cleaved caspase 3, PARP, cleaved PARP, and control GAPDH protein levels were detected by western blotting, and protein bands were quantified by Image J (*n* = 3). (**d-f**) Cells attached to 12-well plates were incubated with 5 µM amiodarone for different periods of time (6, 12, or 24 h). The cleaved caspase 3, PARP, cleaved PARP, and control GAPDH protein levels were detected by western blotting, and protein bands were quantified by Image J (*n* = 3). (**g**) Cells attached to 96-well plates were incubated with different concentrations of Z-VAD-FMK (12.5, 25, 50, or 100 µM), and cells incubated with 0.1% DMSO were used as the control group. Twenty-four hours later, cell viability was determined through the MTT assay (*n* = 4). (**h**) The cells were pretreated with different concentrations of Z-VAD-FMK for 1 h and then incubated with 5 µM amiodarone for another 24 h. Cell death was measured by LDH release (*n* = 4). (**i-j**) The cells were pretreated with or without 20 µM artemisinin for 1 h and then incubated with 5 µM amiodarone for another 24 h. The protein expression levels were detected by western blotting, and protein bands were quantified by Image J (*n* = 3)

The western blotting results revealed that amiodarone increased the protein expression levels of the apoptosis biomarkers cleaved caspase-3 and cleaved poly (ADP-ribose) polymerase (PARP), and the protein ratio of cleaved PARP to PARP in a concentration- and time-dependent manner (Fig. 2a-f). LDH assay revealed that preincubation with the pancaspase inhibitor Z-VAD-FMK at a concentration of 25 µM for 1 h markably inhibited the amiodarone-induced release of LDH in the medium by 19% (Fig. 2h). The western blotting results revealed that pretreatment with 20 µM artemisinin for 1 h attenuated the increase in the protein ratio of cleaved PARP to PARP caused by amiodarone (Fig. 2i-j). These findings suggest that artemisinin confers cytoprotection to D407 cell cultures against amiodarone-induced apoptotic cell death.

### Artemisinin conferred cytoprotection towards amiodarone-induced cell death in relation to AMPK activation

**Fig. 3:**
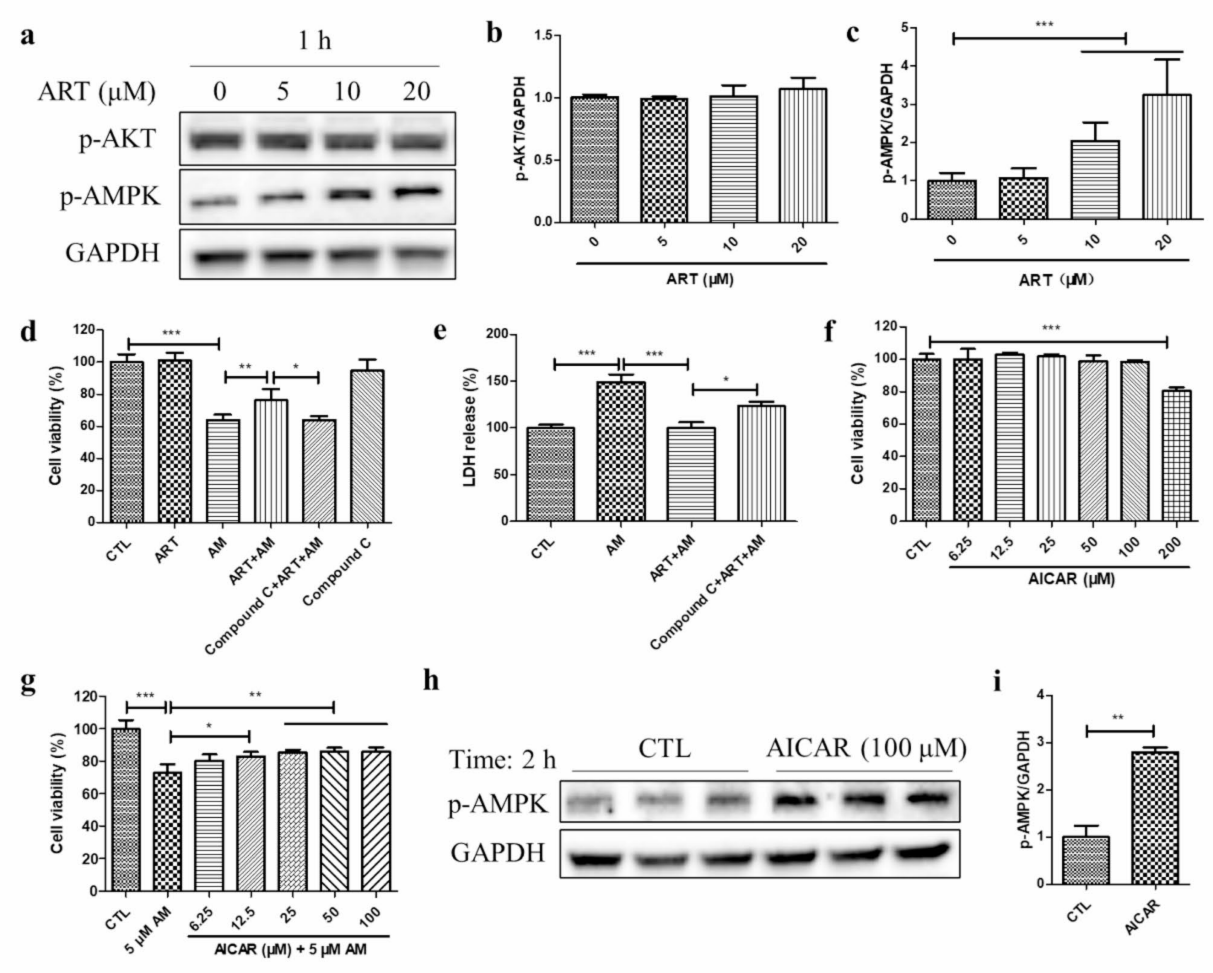
Artemisinin-mediated AMPK activation was linked to its cytoprotective effect on amiodarone-induced D407 cell death. (**a-c**) D407 cell cultures were treated with different concentrations of artemisinin (5, 10, or 20 µM) for 1 h; thereafter, the p-AKT, p-AMPK, and the control GAPDH protein levels were detected by western blotting, and protein bands were quantified by Image J (*n* = 3). (**d-e**) The cells were treated with or without 1 µM Compound C for 30 min, followed by 20 µM artemisinin for 1 h and 5 µM amiodarone for another 24 h. Cell viability was determined by the MTT assay (*n* = 3), and cell death was detected through the LDH cytotoxicity assay (*n* = 4). (**f**) The cells were treated with or without different concentrations of AICAR from 6.25 to 200 µM for 24 h, and cell viability was measured by the MTT assay (*n* = 4). (**g**) The cells were pretreated with or without AICAR at concentrations ranging from 6.25 to 100 µM for 2 h and then incubated with 5 µM amiodarone for another 24 h. Cell viability was determined by the MTT assay (*n* = 4). (**h-i**) Cells attached to 12-well plates were treated with or without 100 µM AICAR for 2 h, the p-AMPK and GAPDH protein levels were detected by western blotting, and protein bands were quantified by Image J (*n* = 3)

The western blotting results revealed that artemisinin concentration-dependently increased the level of phosphorylated AMPKɑ (p-AMPK) but not the level of phosphorylated AKT (p-AKT) (Fig. 3a-c). Meanwhile, artemisinin did not increase the total AKT and AMPK protein levels (Supplementary Fig. 1). This finding suggests that artemisinin activates the AMPK signalling pathway in D407 cells. The AMPK inhibitor Compound C and the AMPK activator AICAR were used to further confirm whether AMPK activation participates in the cytoprotective effect of artemisinin on amiodarone-induced cell death in D407 cell cultures. Figure 3d-e showed that pretreatment with Compound C significantly reversed the cytoprotective effect of artemisinin, indicating that AMPK activation is required for artemisinin-mediated protection of D407 cells from amiodarone-induced cell death. Moreover, the MTT assay results showed that AICAR pretreatment suppressed the amiodarone-induced decrease in cell viability in a concentration-dependent manner (Fig. 3g). These findings further support that AMPK activation is involved in the cytoprotective effect of artemisinin. The increase in p-AMPK level after AICAR treatment was confirmed by western blotting (Fig. 3h-i).

### Artemisinin-mediated AMPK activation in D407 cell cultures was dependent on upstream CaMKK2 but not LKB1

**Fig. 4:**
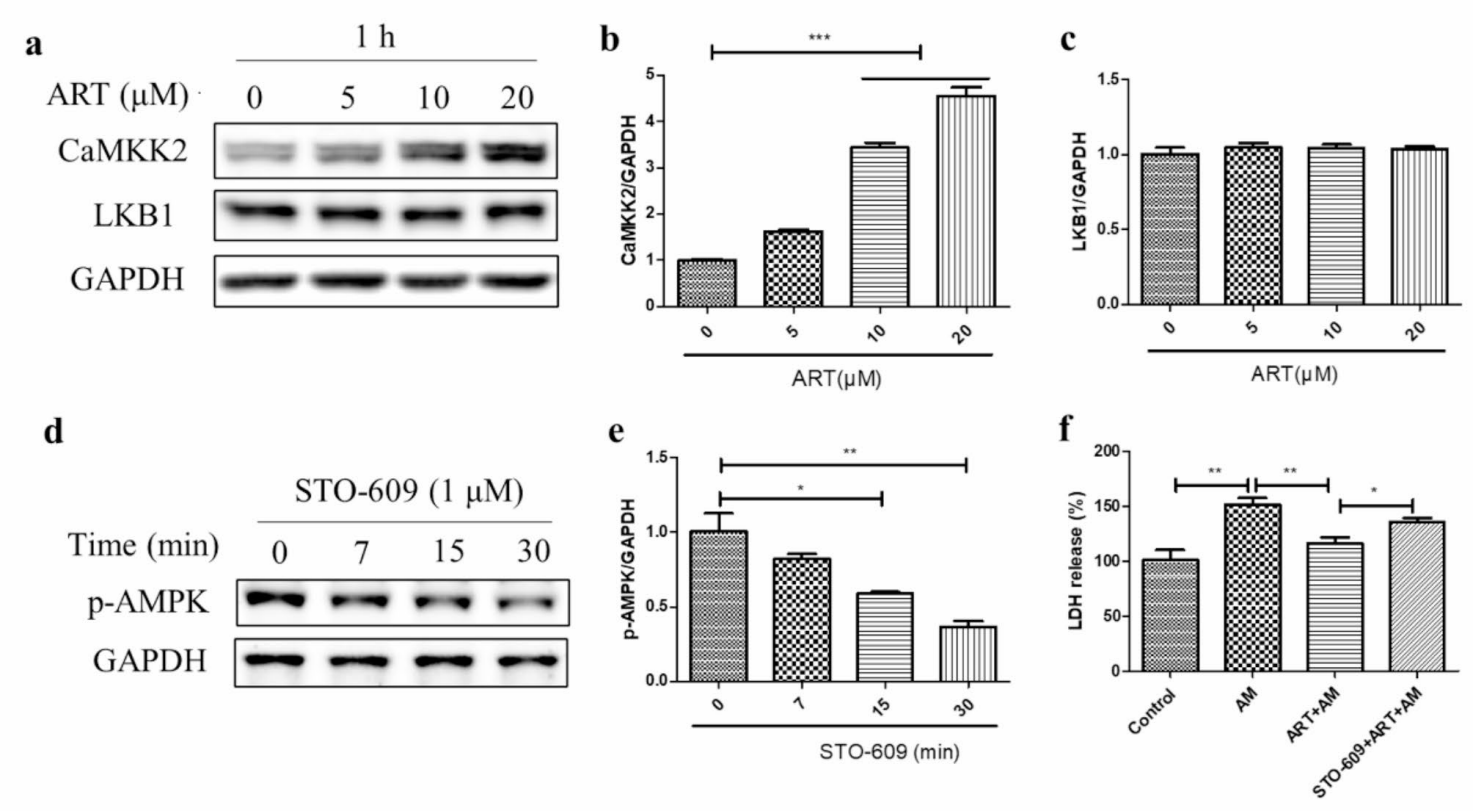
CaMKK2 was involved in the cytoprotective effect of artemisinin on amiodarone-induced cell death in D407 cell cultures. (**a-c**) The cell cultures were incubated with various concentrations of artemisinin for 1 h. The protein expression levels were detected by western blotting, and protein bands were quantified by Image J (*n* = 3). (**d-e**) The cell cultures were incubated with 1 µM STO-609 for 7, 15, or 30 min. The p-AMPK and GAPDH protein levels were detected by western blotting, and protein bands were quantified by Image J (*n* = 3). (**f**) The cell cultures were incubated with or without 1 µM STO-609 for 30 min, followed by 20 µM artemisinin for 1 h and 5 µM amiodarone for another 24 h. Cell death was measured by LDH release (*n* = 4)

Since artemisinin activates AMPK, we sought to investigate whether upstream CaMKK2 and LKB1 protein levels were altered in D407 cell cultures after treatment with different concentrations of artemisinin. Figure 4a-c revealed that artemisinin concentration-dependently increased the CaMKK2 protein level by 1.6-fold to 4.5-fold after 1 h of incubation but did not affect the LKB1 protein level (Fig. 4a-c). This finding suggests that artemisinin selectively activates CaMKK2, but not LKB1, in D407 cells. As expected, incubation of D407 cells with the CaMKK2 inhibitor STO-609 (1 µM) resulted in a 20–60% decrease in the p-AMPK level in a time-dependent manner (Fig. 4d-e). Moreover, the cytoprotective effect of artemisinin on amiodarone-induced cell death was reversed by STO-609 pretreatment in D407 cell cultures, which reduced the cytoprotective effect by 18% (Fig. 4f). These results indicate that CaMKK2 mediates the AMPK activation and the cytoprotective effect of artemisinin on amiodarone-induced cell death in D407 cell cultures.

### The cytoprotective effect of artemisinin on amiodarone-induced cell death was linked to the upregulation of Nrf2

**Fig. 5:**
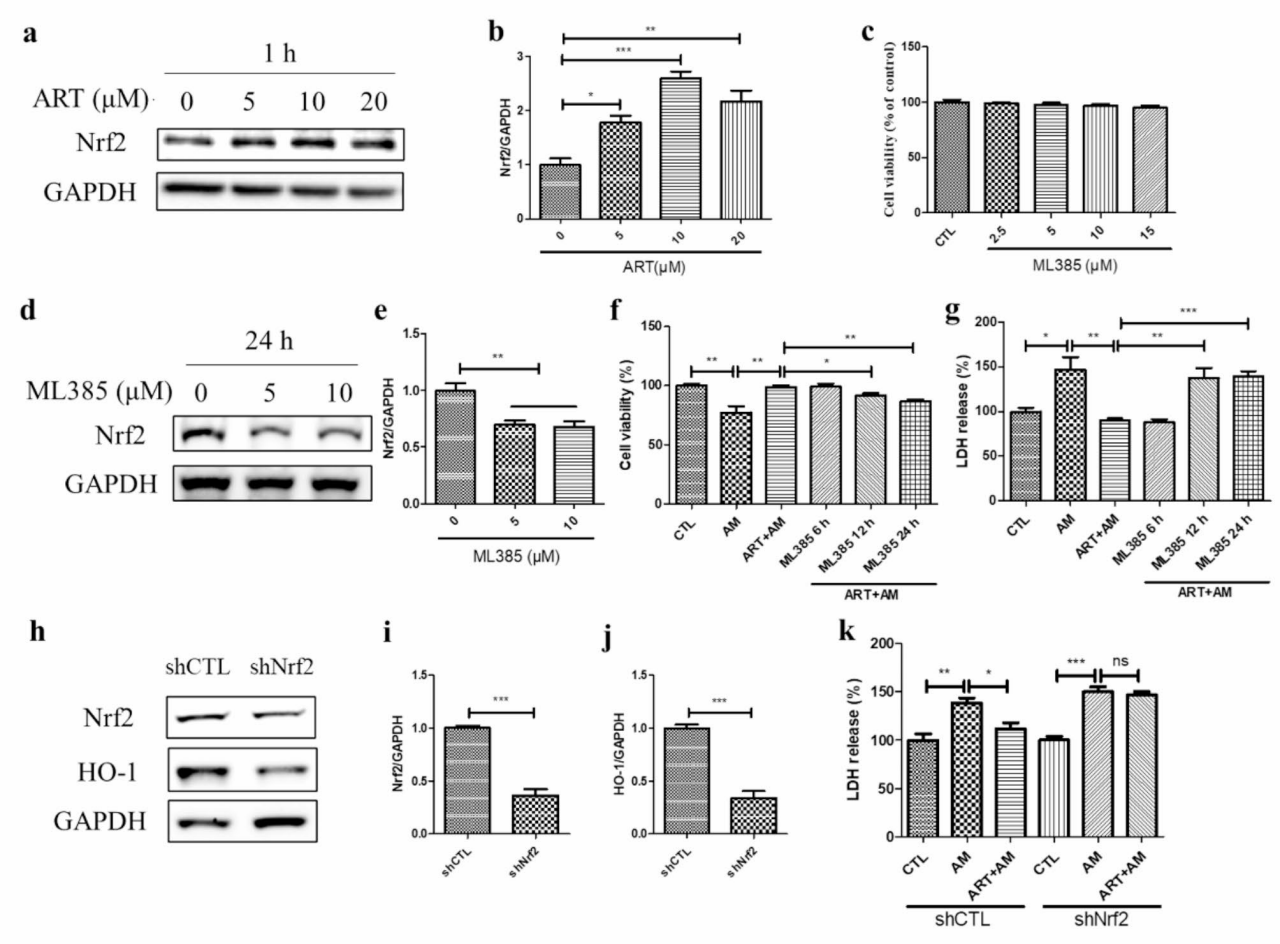
The Nrf2 pathway was involved in the cytoprotective effect of artemisinin on amiodarone-induced cell death in D407 cell cultures. (**a-b**) The cells were treated with artemisinin at various concentrations for 1 h. The Nrf2 and GAPDH protein levels were detected by western blotting, and protein bands were quantified by Image J (*n* = 3). (**c**) The cell cultures were incubated with or without the Nrf2 inhibitor ML385 at concentrations ranging from 2.5 to 15 µM for 24 h, and cell viability was measured using MTT (*n* = 4). (**d-e**) Cells attached to 12-well plates were treated with 5 or 10 µM ML385 for 24 h. The Nrf2 and GAPDH protein levels were detected by western blotting, and protein bands were quantified by Image J (*n* = 4). (**f-g**) The cell cultures were pretreated with 5 µM ML385 for 6, 12, or 24 h, followed by 20 µM artemisinin for 1 h and 5 µM amiodarone for another 24 h. Cell viability was determined by the MTT assay (*n* = 4), and cell death was detected through the LDH cytotoxicity assay (*n* = 4). (**h-j**) Cells attached to 24-well plates were transfected with control shRNA (shCTL) or Nrf2 shRNA (shNrf2) plasmids (0.5 µg/well) for 72 h. The Nrf2, HO-1, and GAPDH protein levels were detected by western blotting, and protein bands were quantified by Image J (*n* = 3). (**k**) Cells transfected with shCTL or shNrf2 plasmids were attached to 96-well plates, and pretreated with artemisinin for 1 h, then treated with amiodarone for another 24 h and processed for LDH assay (*n* = 4)

To elucidate the antioxidant effect of artemisinin, we examined the protein expression level of Nrf2, a major regulator of oxidative stress, that plays an antioxidant role by initiating the transcription of downstream antioxidant enzymes [33]. As shown by the western blotting results, the Nrf2 protein level increased after incubation with artemisinin (5, 10, or 20 µM ) in D407 cell cultures (Fig. 5a-b). This finding suggests that artemisinin activates the Nrf2 signalling pathway in D407 cells. To further confirm the role of Nrf2 in mediating the cytoprotective effect of artemisinin, we used the Nrf2 inhibitor ML385 and Nrf2 knockdown plasmids. According to the results of western blotting, ML385 pretreatment significantly inhibited the protein expression of Nrf2 at 5 and 10 µM after incubation for 24 h (Fig. 5c-e). Moreover, pretreatment with 5 µM ML385 for 12–24 h abolished the cytoprotective effect of artemisinin on amiodarone-induced cell death, as shown by the LDH cytotoxicity assay (Fig. 5f-g). These results indicate that Nrf2 activation is required for the ability of artemisinin to protect D407 cells from amiodarone-induced cell death. Furthermore, the western blotting results revealed that Nrf2 knockdown downregulated the expression of HO-1 (Fig. 5h-j), which is a well-known downstream gene of Nrf2 [34, 35]. Consequently, the knockdown of Nrf2 blocked the cytoprotective effect of artemisinin on amiodarone-induced cell death, as shown by the LDH cytotoxicity assay (Fig. 5k). These results indicate that Nrf2 activation mediates the cytoprotective effect of artemisinin on amiodarone-induced cell death in D407 cell cultures.

### Artemisinin-mediated cytoprotection was linked to the upregulation of HO-1 protein expression and AMPK activation in D407 cell cultures

**Fig. 6:**
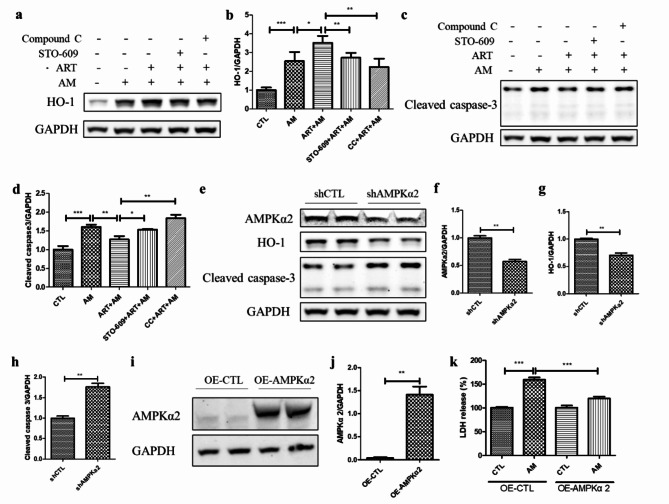
AMPK-mediated activation of the Nrf2/HO-1 pathway was linked to the cytoprotective effect of artemisinin on amiodarone-induced apoptosis in D407 cell cultures. (**a-d**) The cell cultures were pretreated with 1 µM STO-609 for 30 min or 1 µM Compound C for 30 min, followed by 20 µM artemisinin for 1 h and 5 µM amiodarone for another 24 h. The HO-1, cleaved caspase-3, and GAPDH protein levels were detected by western blotting, and the protein bands were quantified by Image J (*n* = 3). (**e-h**) Cells attached to 12-well plates were transfected with shCTL or AMPKɑ2 shRNA (shAMPKɑ2) plasmids (2 µg/well) for 48 h. The AMPKɑ2, HO-1, cleaved caspase-3, and GAPDH protein levels were detected by western blotting, and protein bands were quantified by Image J (*n* = 3). (**i-j**) Cells attached to 24-well plates were transfected with the AMPKɑ2-overexpressing (OE-AMPKɑ2) or control-overexpressing (OE-CTL) plasmids (0.5 µg/well) for 24 h. The AMPKɑ2 and GAPDH protein levels were detected by western blotting, and protein bands were quantified by Image J (*n* = 3). (**k**) The cell cultures transfected with OE-CTL or OE-AMPKɑ2 plasmids for 24 h were attached to 96-well plates and treated with 5 µM amiodarone for 24 h, and processed for the LDH assay (*n* = 4)

The western blotting results revealed that artemisinin pretreatment increased the amiodarone-induced increase in HO-1 protein level by 40% and inhibited the amiodarone-induced increase in cleaved caspase-3 protein level by 20% in D407 cell cultures. However, pretreatment with the CaMKK2 inhibitor STO-609 and the AMPK inhibitor Compound C suppressed the artemisinin-induced increase in HO-1 protein level by 23% and 37%, respectively, and decrease in cleaved caspase-3 protein level by 20% and 44%, respectively (Fig. 6a-d). Furthermore, the 43% knockdown of AMPKɑ2 resulted in a significant decrease in the HO-1 protein level of 29% and a corresponding increase in the cleaved caspase-3 protein level of 75% (Fig. 6e-h). In addition, the overexpression of AMPKɑ2 inhibited amiodarone-induced release of LDH (Fig. 6i-k). These results suggest that the antiapoptotic effect of artemisinin is associated with the AMPK-mediated activation of the Nrf2/HO-1 pathway.

### Artemisinin conferred cytoprotection to ARPE19 cell cultures towards amiodarone-induced cell death via AMPK activation

**Fig. 7:**
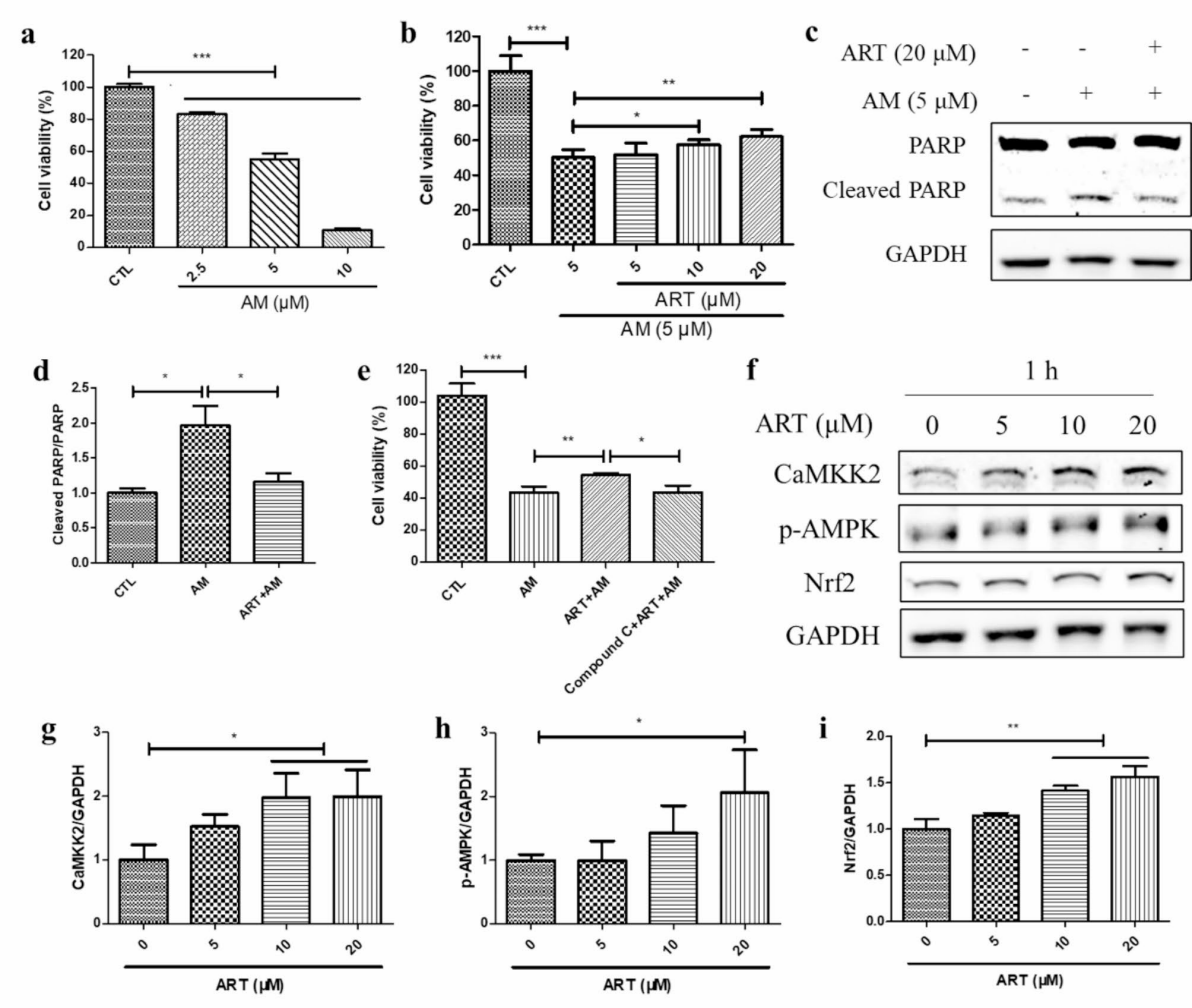
Artemisinin protected ARPE19 cell cultures from amiodarone-induced cell death via AMPK activation. (**a**) ARPE19 cell cultures were treated with or without amiodarone (2.5 ∼ 10 µM) for 24 h. Cell viability was determined by the MTT assay (*n* = 3). (**b**) ARPE19 cell cultures were pretreated with or without artemisinin (5 ∼ 20 µM) for 1 h and then incubated with 5 µM amiodarone for another 24 h. Cell viability was measured by the MTT assay (*n* = 3). (**c-d**) ARPE19 cell cultures were pretreated with or without 20 µM artemisinin for 1 h and then incubated with 5 µM amiodarone for another 24 h. The protein expression levels of PARP, cleaved PARP, and the control GAPDH were detected by western blotting, and protein bands were quantified by Image J (*n* = 3). (**e**) ARPE19 cell cultures were treated with or without 1 µM Compound C for 10 min, followed by 20 µM artemisinin for 1 h and 5 µM amiodarone for 24 h. Cell viability was determined by the MTT assay (*n* = 3). (**f-i**) ARPE19 cell cultures were incubated with artemisinin (0 ∼ 20 µM) for 1 h. The p-AMPK, CaMKK2, Nrf2, and GAPDH protein levels were detected by western blotting, and protein bands were quantified by Image J (*n* = 3)

As indicated by the MTT assay results, treatment with 2.5 to 10 µM amiodarone induced decrease in viability of ARPE19 cell cultures as compared with that of control cells treated with 0.1% DMSO. However, artemisinin pretreatment significantly conferred cytoprotection towards the amiodarone-induced decrease in cell viability in a concentration-dependent manner (Fig. 7a-b). This finding suggests that artemisinin protects ARPE19 cells from amiodarone-induced cell death. According to the results of western blotting, pretreatment with artemisinin attenuated the increase in the protein ratio of cleaved PARP to PARP induced by amiodarone, which supports the cytoprotective effect of artemisinin. (Fig. 7c-d). However, the cytoprotection of artemisinin was suppressed by pretreatment with Compound C, which reduced viability of ARPE19 cells (Fig. 7e). In addition, the western blotting results revealed that the p-AMPK, CaMKK2, and Nrf2 protein levels were increased after ARPE19 cell cultures were treated with artemisinin, suggesting that the CaMKK2/AMPK/Nrf2 pathway was activated (Fig. 7f-i). These results indicate that the cytoprotective effect of artemisinin is mediated by the AMPK/Nrf2 pathway.

### Artemisinin alleviated amiodarone-induced damage to primary human RPE cell cultures, retinal dysfunction, and pathological changes in mouse retinal tissue related to AMPK activation

**Fig. 8:**
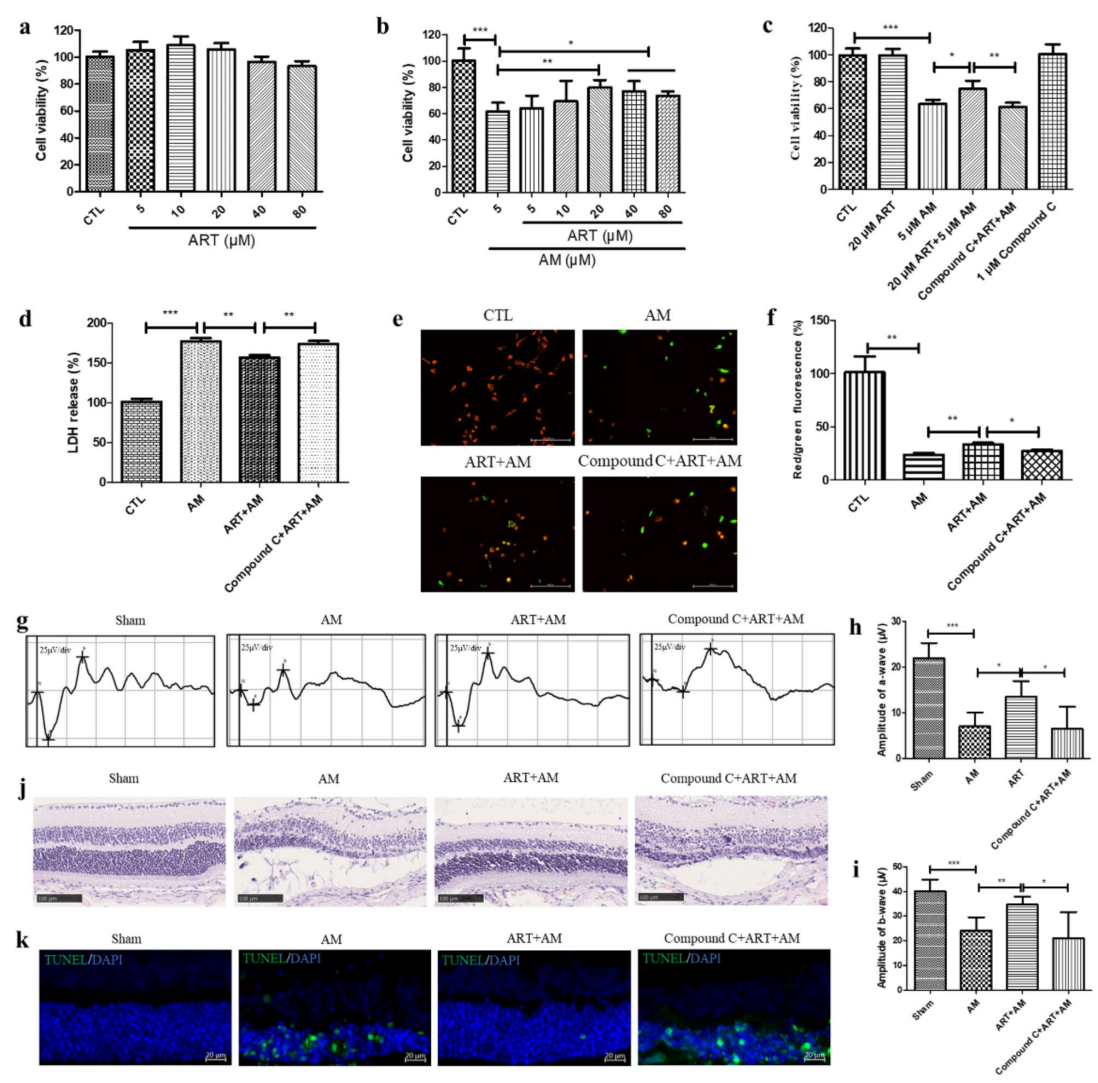
Protective effect of artemisinin against amiodarone-induced cell death in primary human RPE cells and retinal injury in mice in relation to AMPK. (**a**) Primary human RPE cells were treated with 0.1% DMSO (control) or artemisinin (5 ∼ 80 µM) for 24 h. Cell viability was determined by the MTT assay (*n* = 4). (**b**) Cell cultures were pretreated with or without artemisinin (5 ∼ 80 µM) for 1 h and then incubated with 5 µM amiodarone for another 24 h. Cell viability was determined by the MTT assay (*n* = 4). (**c-f**) Cell cultures were treated with or without 1 µM Compound C for 10 min, followed by 20 µM artemisinin for 1 h and 5 µM amiodarone for 24 h. Cell viability was determined by the MTT assay (*n* = 4) (**c**). Cell death was detected by the LDH cytotoxicity assay (*n* = 4) (d). The MMP was measured by using JC-1 dye (*n* = 4) (**e-f**). Green and red fluorescence was observed under a fluorescence microscope, and the fluorescence was quantified using a fluorescence microplate reader. The scale bar is 200 μm. (**g**) Representative photopic 3.0 ERG images of each group. (**h-i**) Photopic 3.0 ERG a- and b-wave amplitudes were analysed (*n* = 5). (**j**) Representative images of HE-stained sections from each group. The scale bar is 100 μm. (**k**) Representative images of the ONL in retinal tissue after TUNEL and 2-(4-Amidinophenyl)-6-indolecarbamidine dihydrochloride (DAPI) double staining. The scale bar is 20 μm

The results of the MTT assay revealed that artemisinin (5 ∼ 20 µM) did not influence the viability of primary human RPE cells (Fig. 8a) but protected cells from the amiodarone-induced decrease in viability in a concentration-dependent manner (Fig. 8b). However, Compound C pretreatment reversed the artemisinin-mediated inhibition of decrease in cell viability, increase in LDH release, and decrease in the MMP induced by amiodarone (Fig. 8c-f). These results support the cytoprotective effect of artemisinin is in accordance with the results obtained in D407 and ARPE19 cell cultures.

In the mouse focal ERG assay, the amplitudes of a-wave and b-wave in the photopic 3.0 ERG response were analysed, and the ERG a-wave and b-wave amplitudes were lower in the AM group than in the sham group. Artemisinin significantly alleviated the decreases in the a- and b-wave amplitudes, indicating improved signal processing in the retina. However, Compound C inhibited this effect of artemisinin (Fig. 8g-i). The HE staining results revealed that the retinal pigment epithelium was detached from the ONL and that the thickness of the ONL was lower in the AM group than in the sham group. Artemisinin corrected these histological changes, and Compound C reversed the beneficial effect of artemisinin (Fig. 8j). Cell apoptosis in the retina was measured using the TUNEL assay. TUNEL-positive cells exhibited green fluorescence. The results showed that amiodarone caused photoreceptor cell apoptosis, and this effect could be inhibited by artemisinin. However, artemisinin-conferred cytoprotective effect towards amiodarone-induced photoreceptor apoptosis was suppressed by Compound C (Fig. 8k). These results suggest that artemisinin protects the mouse retina from amiodarone-induced dysfunction and pathological changes by activating the AMPK pathway.

## Discussion

Although the reported incidence of amiodarone-related optic neuropathy is approximately 2%, and approximately 21% of patients with toxic optic neuropathy suffer a decrease in visual acuity after amiodarone cessation, it can have a profoundly detrimental and lasting impact on the patient’s daily life [3]. The in vitro experiments showed that amiodarone could induce a significant increase in ROS level due to an imbalance in the antioxidant capacity of D407 cells [13]. Oxidative stress can in turn induce retinal degeneration, which can cause vision loss and even blindness [36]. The endogenous antioxidant ability of RPE cells can partially protect the retina from degeneration caused by oxidative stress [37]. Therefore, enhancing the oxidative defense of RPE cells with a multifunctional cytoprotective drug may be a novel approach for coping with amiodarone-induced vision loss. In the present study, the cytoprotective effect of artemisinin on amiodarone-induced D407 cell injury was first characterized by demonstrating its beneficial effect on ameliorating the amiodarone-induced decrease in cell viability, increase in LDH release and ROS level, decrease in the MMP, and apoptosis. Second, the upregulation and/or activation of a few signalling protein kinases, such as CaMKK2-dependent AMPK, was found to be related to the cytoprotective effects of artemisinin on amiodarone-induced cell death in D407 cells. This relationship was further confirmed using the AMPK inhibitor Compound C and the AMPK activator AICAR. Moreover, different studies have reported that Nrf2 plays an essential role in the antioxidant response of RPE cells [38–41]. The degeneration of RPE cells and a decrease in visual function were found in Nrf2-deficient mice [38, 42, 43]. Here, artemisinin exerted antioxidant effect by upregulating the protein levels of Nrf2 and downstream HO-1, which was dependent on AMPK activation, thus conferring cytoprotection to RPE cells against amiodarone-induced apoptosis. These findings are in line with those of other studies investigating the cytoprotective mechanisms of natural compounds. For example, diosgenin, a natural steroid with antidiabetic properties, activates the AMPK/Nrf2/HO-1 pathway, which protects against high glucose-induced death of ARPE-19 retinal pigment cell cultures (a model of diabetic retinopathy) [44]. Morin hydrate, a flavonoid isolated from *Maclura tinctoria*,* Maclura pomifera*, and *Psidium guajava* leaves, confers cytoprotection to human RPE cells exposed to cigarette smoke extract by mitigating oxidative stress, ER stress, and lipid accumulation through activation of the AMPK/Nrf2/HO-1 signalling pathway [45]. Salvianolic acid A, a stilbenoid compound from the traditional Chinese herbal medicine *Salvia miltiorrhiza*, protects RPE cell cultures from oxidative stress by activating the Nrf2/HO-1 signalling pathway [40].

RPE cells play a critical role in maintaining photoreceptor function because the RPE cells can ingest and degrade detached photoreceptor discs, thus achieving outer segment renewal [46, 47], and RPE cells can perform all-trans-retinal recycling and 11-cis-retinal delivery for photoreceptors during the retinal visual cycle [48]. Retinal detachment can induce the degeneration of photoreceptor outer segments and photoreceptor cell death via apoptosis [49]. Moreover, the protective effect of Nrf2 against oxidative damage in the retina was identified, and Nrf2 alleviated apoptosis in photoreceptor cells [41]. Retinal pigment epithelial detachment has been reported in patients with amiodarone-associated optic neuropathy [50]. In the present mouse model, amiodarone-induced retinal damage expressed by the separation of the pigment epithelium was observed in HE-stained retinal sections. ERG was used to evaluate amiodarone-induced retinal toxicity because abnormal a-wave and b-wave amplitudes can be detected in the eyes of rats with amiodarone-induced retinal damage [12, 13]. Here, we found that amiodarone decreased a- and b-wave amplitudes in a mouse model. Photoreceptor damage is one of the retinopathies associated with retinal degeneration [51]. The present experimental results further indicate that amiodarone induced photoreceptor cell apoptosis in mice. The ONL contains photoreceptor nuclei, and its thickness is an important biomarker for retinal degeneration. Artemisinin attenuated amiodarone-induced retinal pathological changes, such as detachment of the pigment epithelium and reduced thickness of the ONL, improved amiodarone-induced dysfunction in the retina, and inhibited amiodarone-induced apoptosis in photoreceptor cells.

Interestingly, these protective effects of artemisinin were inhibited by the AMPK inhibitor Compound C, further supporting the hypothesis that AMPK is involved in the cytoprotective effects of artemisinin. In this study, we revealed a link between AMPK and artemisinin-mediated protection against amiodarone-induced retinal injury. This finding is supported by studies indicating that the AMPK activator metformin could protect photoreceptors and the RPE cells from degeneration by enhancing oxidant defence and energy metabolism [21, 41]. The possibility that AMPK-mediated energy metabolism is also involved in the protection of artemisinin is presently unknown and deserves another study.

## Conclusions

**Fig. 9:**
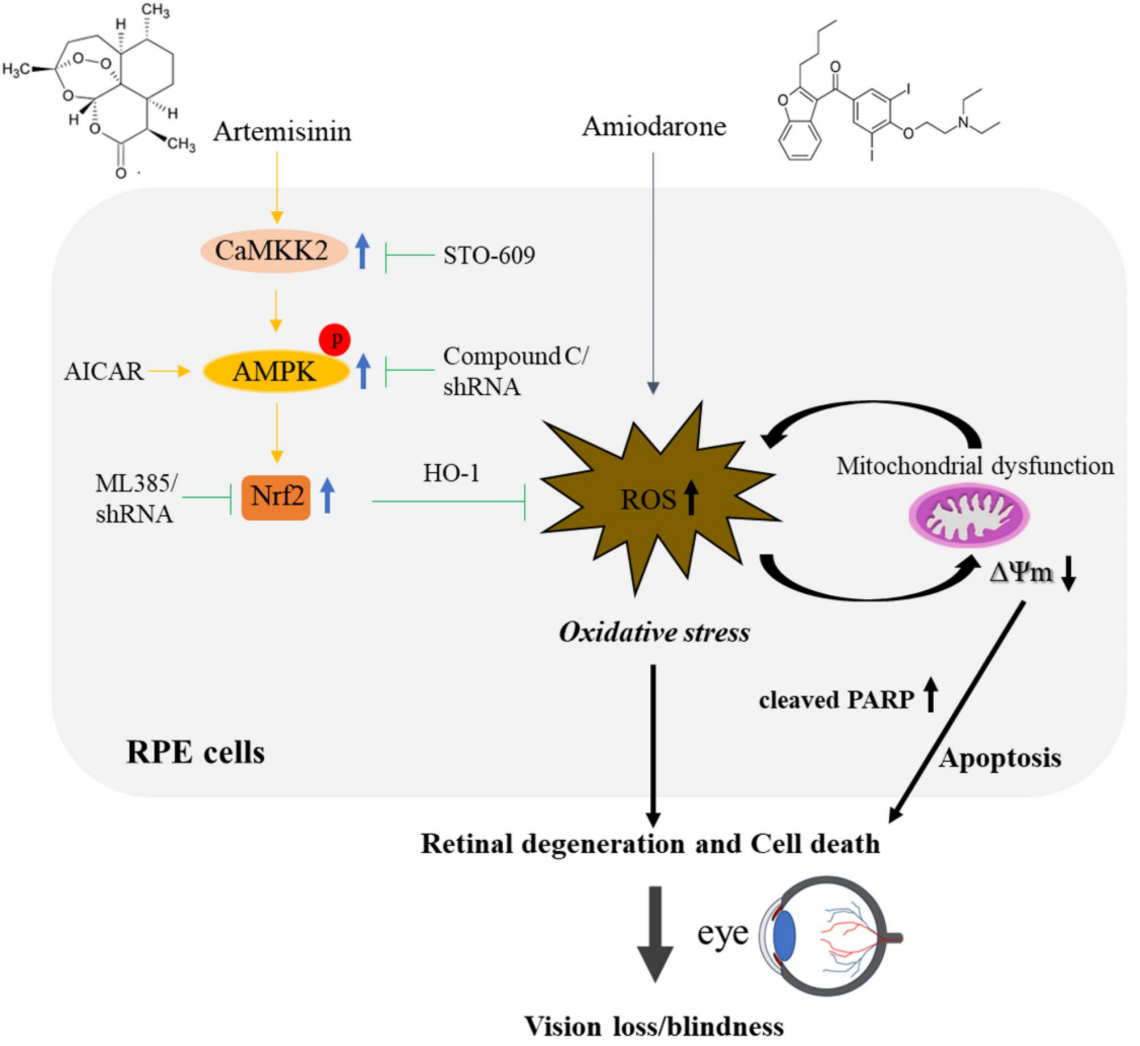
Summary of the signalling mechanisms by which artemisinin confers protection against amiodarone-induced retinal injury. Artemisinin reduced the oxidative damage induced by amiodarone through activation of the CaMKK2/AMPK/Nrf2 pathway, thus reducing the degree of retinal cell death and degeneration. Blue sharp arrow, activated; green blunt arrow, inhibited; yellow sharp arrow, cytoprotective effects; black sharp arrow, pathological events. Abbreviations: CaMKK2, calcium/calmodulin-dependent protein kinase kinase 2; AMPK, adenosine monophosphate-activated protein kinase; Nrf2, nuclear factor erythroid 2-related factor 2; HO-1, heme oxygenase-1; AICAR, 5-aminoimidazole-4-carboxamide ribonucleotide; shRNA, short hairpin RNA; RPE, retinal pigment epithelial; ROS, reactive oxygen species; PARP, poly (ADP-ribose) polymerase

In summary, this study supports the hypothesis that the AMPK activation induced by artemisinin is linked to the activation of the antioxidant master regulator Nrf2, thus conferring cytoprotection to RPE cells towards amiodarone-induced oxidative damage and amiodarone-induced retinal injury in mice (Fig. 9). Considering that oxidative stress is also involved in the toxicity of amiodarone in the kidney, liver, or lung [52–54], it is tempting to propose that artemisinin-mediated AMPK activation and stimulation of Nrf2 and antioxidant signalling, might be potentially useful for the prevention and/or treatment of adverse effects of amiodarone in other organs. The cytoprotective mechanism by which artemisinin enhances the antioxidant capacity of RPE cells via the CaMKK2/AMPK/Nrf2 signalling pathway in the context of amiodarone-induced retinal injury may also provide clues for treating AMD and other retinal degenerative diseases characterized by the loss of photoreceptors or RPE cells.

## Electronic supplementary material

Below is the link to the electronic supplementary material.

Supplementary Material 1

## Data Availability

The datasets used and/or analysed during the current study are available from the corresponding author on reasonable request.

## Abbreviations

RPE: Retinal Pigment Epithelial
LDH: Lactate Dehydrogenase
ROS: Reactive Oxygen Species
MMP: Mitochondrial Membrane Potential
PARP: Poly (ADP-Ribose) Polymerase
AMPK: Adenosine Monophosphate-Activated Protein Kinase
p-AMPK: Phosphorylated AMPKɑ
CaMKK2: Calcium/Calmodulin-Dependent Protein Kinase Kinase 2
Nrf2: Nuclear Factor Erythroid 2-Related Factor 2
AMPKɑ: AMPK ɑ-subunit
LKB1: Liver Kinase B1
HO-1: Heme Oxygenase-1
AMD: Age-Related Macular Degeneration
AICAR: 5-Aminoimidazole-4-Carboxamide Ribonucleotide
OD: Optical Density
BCA: Bicinchoninic Acid
CST: Cell Signaling Technology
SAB: Signalway Antibody
shRNA: short hairpin RNA
ERG: Electroretinography
ONL: Outer Nuclear Layer
INL: Inner Nuclear Layer
HE: Hematoxylin-Eosin
TUNEL: TdT-mediated dUTP Nick-end Labelling
AM: Amiodarone
ART: Artemisinin
CTL: Control
p-AKT: phosphorylated AKT
shCTL: control shRNA
shNrf2: Nrf2 shRNA
shAMPKɑ2: AMPKɑ2 shRNA
OE-AMPKɑ2: AMPKɑ2-overexpressing
OE-CTL: Control-Overexpressing
DAPI: 2-(4-Amidinophenyl)-6-indolecarbamidine dihydrochloride
