# Supplementary material for: Artemisinin conferred cytoprotection to human retinal pigment epithelial cells exposed to amiodarone-induced oxidative insult by activating the CaMKK2/AMPK/Nrf2 pathway
Source: J Transl Med. 2024 Sep 16;22:844. doi: 10.1186/s12967-024-05593-x (PMC11403947; doi:10.1186/s12967-024-05593-x)
Supplement: Supplementary file 1 — Supplementary Material 1 [file 12967_2024_5593_MOESM1_ESM.docx]

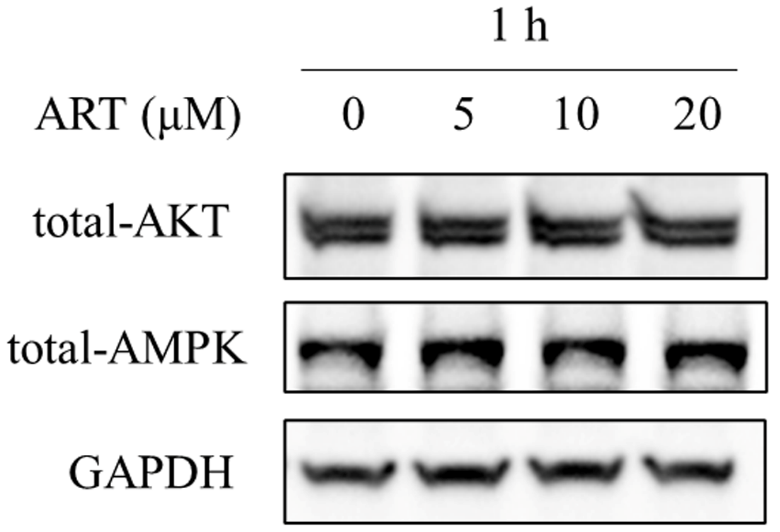


Supplementary Figure 1. Artemisinin does not affect the expression of total AKT and AMPK protein levels. D407 cell cultures were treated with different concentrations of artemisinin (5, 10, or 20 μM) for 1 h, and the protein levels of the total-AKT (CST, #4691), total-AMPK (CST, #2603), and control GAPDH (SAB, #40493) were detected by western blotting. A representative western blotting image depicting the levels of total AKT, total AMPK, and GAPDH proteins is shown.
